# Altered functional connectivity in persistent developmental stuttering

**DOI:** 10.1038/srep19128

**Published:** 2016-01-08

**Authors:** Yang Yang, Fanlu Jia, Wai Ting Siok, Li Hai Tan

**Affiliations:** 1 Neuroimaging Laboratory, School of Biomedical Engineering, Shenzhen University Health Science Center, Shenzhen, China; 2 Shenzhen Institute of Neuroscience, Shenzhen, China; 3 Guangdong Key Laboratory of Biomedical Information Detection and Ultrasound Imaging, Shenzhen, China; 4 School of Humanities, University of Hong Kong, Pokfulam Road, Hong Kong

## Abstract

Persistent developmental stuttering (PDS) is a speech disorder that impairs communication skills. Despite extensive research, the core causes of PDS are elusive. Converging evidence from task-induced neuroimaging methods has demonstrated the contributions of the basal ganglia and the cerebellum to PDS, but such task-state neuroimaging findings are often confounded by behavioral performance differences between subjects who stutter and normal controls. Here, using resting-state functional magnetic resonance imaging, we investigated functional connectivity within cerebellar-cortical and basal ganglia-thalamocortical networks in 16 adults who stutter and 18 age-matched fluent speakers. Seed-to-voxel analysis demonstrated that, compared to controls, adults who stutter showed alternations in functional connectivity of cerebellum to motor cortex as well as connectivity among different locals within cerebellum. Additionally, we found that functional connectivity within cerebellar circuits was significantly correlated with severity of stuttering. The alternations of functional connectivity within basal ganglia-thalamocortical networks were identified as the reduced connectivity of the putamen to the superior temporal gyrus and inferior parietal lobules in adults who stutter. The abnormalities of resting state functional connectivity are assumed to affect language planning and motor execution critical for speaking fluently. Our findings may yield neurobiological cues to the biomarkers of PDS.

Fluent speech is important for human communication, but difficult for the 1% of the adult population who have persistent developmental stuttering (PDS)^1^. Stuttering is a neurogenetic speech disorder characterized by involuntary repetitions, and/or prolongations, and/or blocking of sounds, syllables or words^2^. Task-related functional magnetic resonance imaging (fMRI) studies have identified a number of brain regions associated with PDS including auditory-associated areas^3^,^4^, premotor areas^3^,^5^,^6^,^7^,^8^, the basal ganglia^9^,^10^, and the cerebellum^7^,^8^,^9^. However, such task-state neuroimaging findings are often confounded by behavioral performance differences between subjects who stutter and normal controls. For example, speaking rates are different for stutterers and normal controls, which significantly influence brain activity^11^, implying the large contribution of task performance to the findings of neural abnormalities identified by task-based studies in PDS. This limitation can be overcome by using resting-state fMRI, a powerful tool for understanding neurophysiological mechanisms by measuring brain activity while the subject is in a task-free state^12^. Resting state functional connectivity (RSFC) is an index of synchronization of neural activity that represents the correlations of spontaneous blood oxygen level dependent (BOLD) fluctuation^13^. Previous studies have shown that RSFC could reliably predict task-response activity^14^ and individual differences in behavior^15^, indicating that RSFC carries meaningful neurobiological information. Critically, resting state fMRI circumvents the limitations of task requirements for patient subjects who are incapable of carrying out tasks accurately as normal population due to cognitive or physical dysfunction. Hence, RSFC has great promise for clinic applications, such as exploring the neural signatures of PDS. The abnormalities of RSFC are highly linked to PDS itself, rather than the task performance and are thus thought to reflect the core causes of stuttering^16^. However, in contrast to the extensive knowledge of neural mechanisms revealed by task-based neuroimaging studies, far less is known about RSFC of PDS. Previous studies have demonstrated atypical RSFC within auditory-motor and basal ganglia-thalamocortical networks in children with PDS^17^ and sensorimotor and default-mode networks in adults with PDS^18^.

The cerebellum and basal ganglia are important subcortical structures that mediate cognition, motor and emotion processing via interacting with cerebral cortex. The cerebellum, one of neural regions implicated in stuttering^19^, has been shown to play an important role in enabling fluent speaking for persons who stutter^6^. Using independent component analysis (ICA) analysis, one study revealed that RSFC patterns of the cerebellum are different between people who stutter and fluent speakers^16^. However, this ICA analysis can hardly tell the specific regions that are abnormally connected with the cerebellum and reveal the anticorrelation among individual regions which is a prominent feature of spontaneous activity during rest^20^.Furthermore, a recent diffusion tensor imaging (DTI) study demonstrated that very young children with PDS showed abnormal fractional anisotropy (FA) in the bilateral cerebellum relative to age-matched peers^21^, implying the structural connectivity abnormalities in PDS.

The dysfunction of basal ganglia is also thought to lead to stuttering^22^. The activity of basal ganglia during speech tasks was found to be positively correlated with stuttering rate^3^ and severity of stuttering^23^. Using structural equation modeling (SEM), effective connectivity analysis of task-evoked fMRI data revealed alternated connectivity of the basal ganglia to the temporal gyrus and pre-supplemental motor area (SMA) in stuttering subjects relative to controls. Another resting state fMRI study revealed the alternation of RSFC between the basal ganglia and SMA in children with PDS^17^. However, whether such abnormalities are exhibited in adults who stutter have not been examined.

Because several functions of the cerebellum and the basal ganglia are critical to fluent speaking and thus they are candidates of stuttering^17^,^24^, research on the RSFC of the cerebellum and basal ganglia may yield neurobiological cues to the causes of stuttering. Here, using a seed-driven method in resting-sate fMRI, we examined functional connectivity within cerebellar-cortical and basal ganglia-thalamocortical networks in adults who stutter, as compared with age-matched fluent speakers.

## Results

### The role of cerebellar-cortical networks in PDS

**Figure 1:**
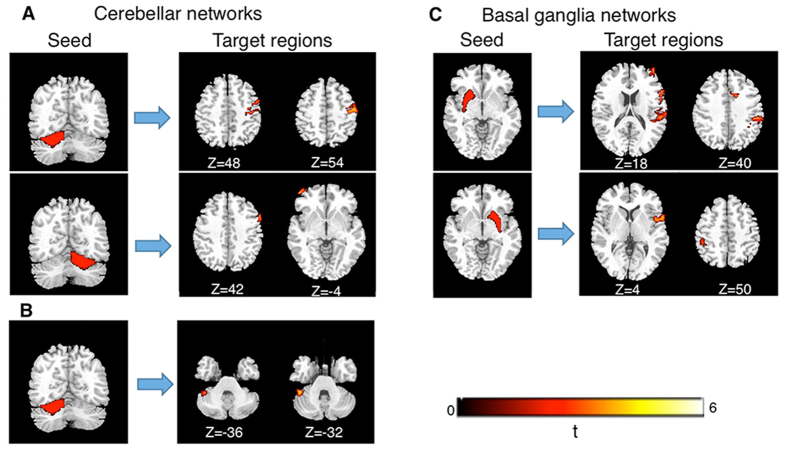
Group differences in RSFCs within cerebellar and basal ganglia networks. Thresholds are set at voxelwise (p < .005), uncorrected, cluster level (p < 0.03), corrected, (k > 64), and Monte Carlo simulation. (**A**) Significant stronger connectivity based on the cerebellar lobule VI seeds in controls compared to subjects who stutter (**B**) Significant stronger connectivity based on the cerebellar lobule VI seed in subjects who stutters compared to controls; (**C**) Significant stronger connectivity based on the putamen seeds in controls compared to subjects who stutter. Statistical Parametric Map (SPM) is overlaid on the corresponding T1 image (in grey scale). L, left hemisphere; R, right hemisphere.

We found abnormal RSFCs between cerebellar seeds and frontal regions as well as distinct locals within the cerebellum (Fig. 1, Table 1). Specifically, the RSFC between the left lobule VI and right motor areas (Brodmann’s areas, BA4/6) was negative in subjects who stutter (r = –0.09, p = 0.008), but positive in controls (r = 0.12, p = 0.007). Similarly, the subjects who stutter exhibited negative RSFC between the right cerebellum lobule VI and bilateral middle frontal gyrus (left: r = –0.09, p = 0.008; right: r = –0.06, p = 0.04) whereas controls showed significant positive connectivity (left: r = 0.07, p = 0.02; right: r = 0.08, p = 0.003). In addition to the atypical RSFCs between the cerebellum and cortical regions, the subjects who stutter also exhibited aberrant RSFCs within the cerebellum itself. Compared with controls, stuttering subjects showed weaker positive RSFC between the right lobule VI and left lobule VII (stuttering subjects: r = 0.08, p = 0.02; controls: r = 0.28, p < 0.001; stuttering subjects *vs*. controls: t(32) = –4.51, p < 0.001). In addition, greater positive RSFC between the left lobule VI and left crust 1 was seen in stuttering subjects than in controls (stuttering subjects: r = 0.36, p < 0.001; controls: r = 0.15, p < 0.001; stuttering subjects *vs*. controls: t(32) = 4.15, p < 0.001).

**Figure 2:**
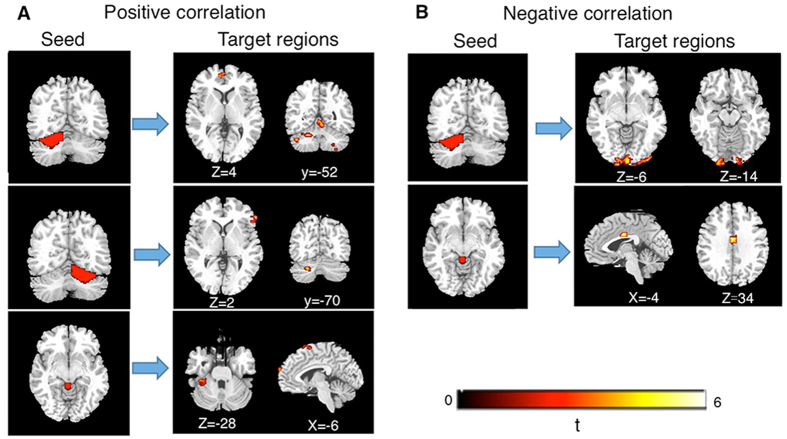
Correlation between cerebellar-cortical RSFCs and severity of stuttering. Thresholds are voxelwise (p < 0.005), uncorrected, cluster level (p < 0.03), corrected, (k > 64), and Monte Carlo simulation. (**A**) Positive correlation between RSFCs and severity of stuttering. (**B**) Negative correlation between RSFCs and severity of stuttering. Statistical Parametric Map (SPM) is overlaid on the corresponding T1 image (in grey scale). L, left hemisphere; R, right hemisphere.

Apart from group comparison, we employed regression analysis using the stuttering severity scores as the regressor to determine whether there is a relationship between stuttering severity and RSFCs in cerebellar loops (Fig. 2, Table 2). Through this analysis, we observed the severity of stuttering was positively correlated with RSFCs between the right lobule VI and the right inferior frontal gyrus (BA45) (r = 0.85, p < 0.001) and between the vermis III and the right superior frontal gyrus (BA6) (r = 0.76, p < 0.001). Within the cerebellum, the RSFCs between the left lobule VI and right lobule VIII and between the right lobule VI and left crust I showed a positive correlation with severity of stuttering (former: r = 0.78, p < 0.001; latter: r = 0.75, p = 0.001). Differing from the above patterns of positive correlations, we observed that several RSFCs of the cerebellum were negatively correlated with severity of stuttering. The stuttering severity was negatively correlated with RSFCs between the left lobule VI and the left lingual gyrus (r = –0.84, p < 0.001) and between the vermis III and the anterior cingulate gyrus (BA24) (r = –0.88, p < 0.001).

### The role of basal ganglia-thalamocortical networks in PDS

Since atypical RSFC in basal ganglia-thalamocortical networks has previously been detected in childhood PDS, we tested whether these networks were dysfunctional in adults with PDS (Fig.1C, Table 3). The results showed that stuttering subjects exhibited significantly negatively correlation between the left putamen and the right medial frontal gyrus/SMA(r = –0.13, p < 0.001), which was absent in controls (r = 0.04, p = 0.102). In addition, our analysis indicated that, compared with controls, subjects who stutter failed to show positive RSFC between the left putamen and the right superior temporal gyrus (stuttering subjects: r = 0.02, p = 0.32; controls: r = 0.22, P < 0.001) and showed a weaker positive RSFC between the right putamen and the right superior temporal gyrus (stuttering subjects: r = 0.26, p < 0.001; controls: r = 0.43, p < 0.001; stuttering subjects *vs*. controls: t(32) = –4.59, p < 0.001). Finally, the connectivity between the putamen and inferior parietal lobule (BA40) differed between the group who stutter and the control group. The control speakers showed positive RSFC between the right putamen and the left inferior parietal lobule (r = 0.08, p = 0.008,) whereas those who stutter exhibited negative RSFC in this connectivity (r = –0.08, p = 0.01). Similarly, the subjects who stutter showed significantly negative RSFC between the left putamen and the right inferior parietal lobule (r = –0.14, p < 0.001) while controls did not exhibit a significant correlation (r = 0.03, p = 0.26).

## Discussion

Consistent with a prior resting state fMRI study^16^, we find that adults who stutter exhibited abnormal RSFCs of the bilateral cerebellum relative to fluent speakers. More importantly, our findings further clarified the particular regions involving the right motor and bilateral prefrontal gyrus that were abnormally connected with cerebellum in stuttering subjects. In task-evoked fMRI studies, abnormal activation of the bilateral cerebellum has been reported^25^, which could be normalized by treatment^26^. Cerebellum is known to be recruited to support acquiring new motor sequences^27^, execution of pre-learned motor sequences^28^ and timing of motor^29^. Particularly, studies using resting-state fMRI to parcellate the cerebellum revealed that the bilateral lobule VI are mainly connected with sensorimotor regions^30^. The abnormal functional connectivity between the lobule VI and motor areas in the subjects who stutter is thought to lead a deficit in integrating motor-related regions to execute serial motor during speech production^5^. Beyond the motor function, the RSFC between the cerebellum and prefrontal gyrus has been evidenced in normal people, supporting the view that cerebellum has high level of cognitive function by coupling prefrontal gyrus^31^. Moreover, the anterior prefrontal gyrus has been assumed to be involved in high levels of cognitive processes such as coordination and communication of information for different cognitive operations^32^. Thus, in people who stutter, the abnormal decoupling between the right lobule VI and bilateral prefrontal gyrus may result in problems in executive (cognitive) functioning such as the difficulty in word retrieval^33^. In addition, the results of correlation analysis between RSFCs and severity of stuttering aid to elucidate the roles of cerebellar-cortical networks in stuttering. We find that the RSFC between the right lobule VI and the right inferior frontal gyrus was positively correlated with severity of stuttering. Previous task-induced studies have shown hyperactivity of right frontal regions during speech tasks in people with PDS that has been considered as compensatory efforts^4^,^8^. Functionally, the right inferior frontal gyrus was engaged in inhibition processing of speech act during speech production^34^. Thus, our results imply a functional connectivity basis for compensatory efforts in PDS. We also observed that the RSFC between the vermis III and the left cingulate gyrus was negatively correlated with severity of stuttering. Previous studies demonstrated that the vermis III was uniquely related to stuttering^6^,^19^, but its specific role is unclear. The cingulate gyrus is the neural basis for attentional control^35^, and therefore our results may reflect a progressive lack of functional network development serving the attentional monitoring of inner state in speech in those who stutter more severely, shedding light on the role of vermis III in stuttering.

Different parts of the cerebellum play unique roles in supporting motor functions, cognition, and emotion via integrating different cortical regions^30^,^31^. Speech is a complicated activity involving primary motor control and high-level executive processes and therefore requires synchronization of neuronal firing within distinct regions of the cerebellum. Thus, in PDS, the absent or lower RSFCs between left and right cerebellar regions may reflect the difficulty in integration of cerebellar motor control and high-level execution functions. Additionally, our results indicated that the RSFC between the lobule VI and lobule VIII was correlated with severity of stuttering. Bilateral lobule VI and VIII both correlate with motor/premotor regions^30^ and thus, the motor execution or preparation skills required to integrate distinct functional locales of the cerebellum are related to stuttering.

In line with the findings of children with PDS^17^, we find that stuttering subjects showed abnormal RSFC between the basal ganglia and SMA relative to controls. However, unlike the finding in children with PDS, our results indicated that the right SMA, rather than the left SMA was abnormally connectivity with the putamen in adults with PDS, which could be considered as right-lateralized compensatory mechanisms. Basal ganglia and SMA are key nods of network supporting self-paced motor sequences^36^. The motor function of the basal ganglia has been proposed as inhibition of unwanted competing alternates^37^,^38^ or providing inter timing cues^39^,^40^. The inhibition mechanism of the basal ganglia could explain why the extra RSFC between the putamen and the right SMA is negative in PDS. In consistent with previous effective connectivity and RSFC analysis^17^,^41^, we find that stuttering subjects exhibited reduced RSFC between the bilateral putamen and the right superior temporal gyrus, suggesting an important neural basis of stuttering. The bilateral superior temporal gyrus supports the perception of one’s own voice as a source of feedback for self-monitoring during speech production^42^ and particularly, the right superior temporal gyrus has been proposed to serve rehearsal of rhythmic pattern^43^. In PDS, these changes in functional connectivity between the putamen and superior temporal gyrus may affect sensorimotor integration between auditory feedback and motor control during speech production^44^.The results that adults with PDS showed the disconnection between the basal ganglia and inferior parietal lobule are intriguing that has not been detected in children with PDS, suggesting this abnormality is probably a result of aberrant development. Both the putamen and left inferior parietal lobule are known to be nodes of networks for sequence processing of phonological units critical for fluent speaking^45^,^46^. The left inferior parietal lobule is responsible for temporal analysis of syllables in short-term memory^46^. Thus, for persons who stutter, the impairment in connectivity between the putamen and the left inferior parietal lobule may lead to difficulties in planning of sequential phonological units. Besides, stuttering subjects have additional anticorrelation between the left putamen and right interior parietal lobule. The activation of the right inferior parietal lobule (supramarginal gyrus) was decreased in people who stutter during dysfluent speech production, suggesting its particular role in speech for stuttering subjects^3^. Functionally, the right supramarginal gyrus was found to subserve the lower level of acoustic-phonological processing^47^ that requires the temporal analysis supported by the basal ganglia. Our results suggest that dysfluency in people who stutter is in part due to a failure of coupling the basal ganglia and the inferior parietal lobule which is hypothesized to support phonological processing for speech planning. Future neuroimaging studies are needed to clarify the specific contributions of the inferior parietal lobule to PDS.

In summary, this study has demonstrated that there are alterations of intrinsic interactions in cerebellar networks in PDS, which supports previous observations that the cerebellum plays an important role in PDS. Moreover, we observed abnormal RSFCs within basal ganglia loops in adults with PDS. These findings shed new light on the pathophysiological causes of abnormal speech.

## Method

### Participants

Thirty-four participants were scanned using fMRI: 16 stutterers (14 male and 2 female; mean age = 26.3, range from 21 to 35) and 18 controls (15 male and 3 female; mean age = 24.7, range from 22 to 31). All stuttering participants started stuttering before teenager, and had not underwent treatment during the year prior to this study. The severity of stuttering subjects ranged from very mild to very severe as judged by the Stuttering Severity Instrument-3 (SSI-3)^48^ and the Overall Assessment of the Speaker’s Experience of Stuttering (OASES)^49^. In order to investigate the metalinguistic and cognitive abilities, several linguistic and cognitive tests are conducted on both groups of subjects including rapid number/picture naming, phonological awareness and phonological working memory. All participants were physically healthy and had no history of neurological disease or psychiatric disorder based on their self-report. They were native Chinese speakers and were right-handed as assessed by the handedness inventory^50^ (Table 4). Prior to experiment, inform consent was obtained from each subject. The study was approved by the Institutional Review Board of Beijing MRI Center for Brain Research. The methods were carried out in accordance with the approved guidelines. All experimental protocols were approved by the Institutional Review Board of Beijing MRI Center for Brain Research.

### Imaging acquisition

MRI data were collected on a Siemens 3T Siemens MRI scanner at the Beijing MRI Center for Brain Research of the Chinese Academy of Science. Participants were required to close their eyes and relax without intentional thinking. Functional images were acquired by an 8 minutes scan using a blood oxygen level-dependent (BOLD)-sensitive gradient echo-plane-image (EPI) sequence (TR = 2000 ms, TE = 30 ms, slices thickness = 4 mm, in-plane resolution = 3.4 mm × 3.4 mm and flip angle = 90°). Thirty-three axial slices were collected. High spatial resolution anatomical images were acquired using a T1-weighted, magnetization-prepared rapid acquisition gradient echo (MPRAGE) sequence (TR = 2600 ms, TE = 3.02 ms, slice thickness = 1 mm, in-plane resolution = 1.0 mm × 1.0 mm and flip angle = 8°).

### Data analysis

Image preprocessing and statistical analyses were processed by using SPM8 (http://www.fil.ion.ucl.ac.uk/spm/, Wellcome Department of Cognitive Neurology, University College London, London). fMRI image was corrected for motion, coregistered to the native T1, spatially normalized into the Montreal Neurological Institute (MNI) stereotactic space with a resolution of 2 × 2 × 2 mm cubic voxels, and then smoothed with an isotropic Gaussian kernel of 6 mm full-width at half-maximum.

Functional connectivity analysis was performed by using the CONN-fMRI toolbox for SPM8^51^. A seed-to-voxel connectivity analysis was conducted which computed the correlation of spontaneous BLOD activity between seeds and other voxels of the brain during rest. The cerebellar seeds were selected based on a prior meta-analysis study showing that were associated with stuttering and fluent speaking^19^.The bilateral cerebellum lobule VI and vermis VIII were included. Following the prior research^17^, the putamen was selected as the seeds for basal ganglia-thalamocortical networks. All seed ROIs were created using the automated anatomic labeling (AAL) atlas^52^. The whole anatomical regions were used to avoid researchers-dependent bias and different size of ROIs could be handled by the CONN toolbox. Using the implemented CompCor strategy^53^, the effect of nuisance covariates including fluctuations in BOLD signal from CSF, white matter and their derivatives, as well as the realignment parameter noises were reduced. Data were band-pass filtered (0.008 < f < 0.09 HZ). Bivariate correlation coefficients between the time serial of seeds and the rest voxels in the brain were obtained and then were transformed to Z-scores. Individual functional connectivity maps were put into a random effects analysis for group difference analysis using independent two-sample *t* test. In order to examine the nature of differences in RSFCs, one sample *t* tests were used to examine the coefficient of RSFCs between seeds and target regions in each group of subjects. For reporting purpose, the Z values of connectivity were transformed back to r values.

In addition to between-group difference analysis, whole-brain regression analysis was employed to detect the relationship between RSFC and the severity of stuttering using SSI score as a covariate in stuttering group. To quantify the extent of correlation, we extracted the connectivity coefficients between the seeds and the target regions, which were subject to correlate with the severity score using Pearson correlation analysis. Following previous research^17^, we used a threshold of voxel-wise p < 0.005 uncorrected with cluster size >64 voxels corresponding to p < 0.03 whole brain corrected using Monte Carlo simulation. Brain regions were estimated based on the Talairach atlas^54^.

## Additional Information

**How to cite this article**: Yang, Y. *et al*. Altered functional connectivity in persistent developmental stuttering. *Sci. Rep*. **6**, 19128; doi: 10.1038/srep19128 (2016).

## Figures and Tables

**Table 1:** RSFC alternations within cerebellar-cortical networks in stuttering subjects.

Seed ROI	Target regions	Talairach					
		BA	x	y	z	z score	Cluster size
Stutterers > Controls							
L lobule VI	L superior frontal gyrus	11	–6	50	–19	3.35	70
	L cerebellum crust I		–44	–42	–26	3.95	104
Vermis III	L cuneus	19	–14	–90	31	3.78	98
Controls > Stutterers							
L lobule VI	R precentral gyrus	4	44	–13	50	3.58	368
		6	46	–2	42	3.38	
	R cerebellum crust I		16	–80	–43	4.52	845
	L cerebellum vermis VIII		–2	–71	–28	3.79	
R lobule VI	L lingual gyrus	17	–12	–96	–12	4.01	82
	L middle frontal gyrus	10	–48	54	–6	3.91	67
	R middle frontal gyrus	8	57	14	40	3.62	67
	L cerebellar lobule VII		–6	–73	–30	3.82	264

The location of the maximum pixel values were expressed in the human brain atlas of Talairach. Z-score correspond to the actual maximum pixel value within the brain region from the SPM (Voxelwise p < 0.005, uncorrected, cluster p < 0.03, corrected, k > 64, Monte Carlo simulation). L = left; R = right; BA = Brodmann’s area.

**Table 2:** Correlation between RSFCs within cerebellar-cortical networks and severity of stuttering.

Seed ROI	Target regions	Talairach					
		BA	x	y	z	z score	Cluster size
Positive correlation							
L lobule VI	L precuneus	7	–26	–81	46	3.79	201
	L anterior cingulate	10	–10	48	–2	3.35	74
	L cerebellum lobule VI		–34	–57	–19	4.34	277
	R cerebellum VIII		30	–59	–46	4.18	152
	R cerebellum vermis VI		6	–66	–8	3.71	245
R lobule VI	R inferior frontal gyrus	45	55	35	2	3.70	77
	L cerebellum lobule III		–24	–69	–23	3.55	98
	L cerebellum crust I		–32	–65	–24	3.23	
Vermis III	R superior frontal gyrus	6	12	15	66	4.05	136
	R superior frontal gyrus	10	14	67	19	3.67	72
	L cerebellum/fusiform		–32	–40	–20	3.06	71
Negative correlation							
L lobule VI	R middle occipital gyrus	18	38	–95	3	4.86	369
	R cuneus	18	4	–99	3		468
	L lingual gyrus	17	–16	–98	–12	3.55	
Vermis III	L cingulate gyrus	24	–2	–9	26	5.01	146
	R postcentral gyrus	1	53	–19	56	3.37	92
	R precentral gyrus	4	57	–12	49	3.33	

The location of the maximum pixel values were expressed in the human brain atlas of Talairach. Z-score correspond to the actual maximum pixel value within the brain region from the SPM (Voxelwise p < .005, uncorrected, cluster p < 0.03, corrected, k > 64, Monte Carlo simulation). L = left; R = right; BA = Brodmann’s area.

**Table 3:** RSFC alternations within basal ganglia-thalamocortical networks in stuttering subjects.

Seed ROI	Target regions	Talairach					
		BA	x	y	z	z score	Cluster size
Stutterers > Controls							
L putamen	L superior temporal gyrus	38	–32	24	–23	3.98	93
Controls > Stutterers							
L putamen	R medial frontal gyrus	32	16	12	47	3.95	90
	R SMA	6	8	8	53	2.91	
	R superior frontal gyrus	10	42	57	16	3.77	167
	R middle frontal gyrus	10	38	49	10	2.89	
	R inferior frontal gyrus	47	50	25	–1	3.81	
	R superior temporal gyrus	22	57	6	2	4.19	1269
	R transverse temporal gyrus	42	59	–15	12	3.90	
	R superior temporal gyrus	21	57	–23	0	3.82	209
	R postcentral gyrus	2	48	–29	37	4.05	620
	R inferior parietal lobule	40	51	–37	44	3.75	
	R cingulate	32	16	17	32	3.54	76
	R anterior cingulate	24	12	21	25	3.20	
	R cerebellum		42	–56	–24	3.45	65
R putamen	R superior temporal gyrus	22	55	8	–2	4.03	206
	R inferior frontal gyrus	47	58	21	–6	2.92	
	L fusiform gyrus	37	–44	–40	–18	3.62	224
	L inferior parietal lobule	40	–48	–34	50	3.60	125
	L postcentral gyrus	5	–44	–42	61	2.70	

The location of the maximum pixel values were expressed in the human brain atlas of Talairach. Z-score correspond to the actual maximum pixel value within the brain region from the SPM (Voxelwise p < 0.005, uncorrected, cluster p < 0.03, corrected, k > 64, Monte Carlo simulation). L = left; R = right; BA = Brodmann’s area. SMA = supplemental motor area.

**Table 4:** Demographic characteristics of the two groups.

	Stutterers (n = 16)	Controls (n = 18)	*p*-Value
	Mean(SD)	Mean(SD)	
Age (in years)	26.31 (3.7)	24.77 (2.86)	0.18
Handedness	all right-handed	all right-handed	
Education(years)	14.87 (2.24)	15.22 (2.22)	0.67
SSI-3	24.37 (5.54)	n/a	
OASES	58.38 (12.23)	n/a	
Rapid naming(s)			
Picture	17.06(2)	15.36(1.58)	0.01
Number	13.96(3.09)	11.85(2.20)	0.03
Phonological awareness	25.31(4.57)	25.05(3.87)	0.86
Phonological working memory			
Forward	8.93(1.28)	9.33(1.23)	0.36
Backward	6.06(1.69)	6.89(1.81)	0.18

Independent-Sample *t*-test was used; SD = standard deviation; s = second. n/a = not applicable.
